# Changes in bone density and bone turnover in patients with rheumatoid arthritis treated with rituximab, a B cell depleting monoclonal antibody (HORUS TRIAL)

**DOI:** 10.1186/1471-2474-14-S1-A10

**Published:** 2013-02-14

**Authors:** Mohsen Elshahaly, Gillian Wheater, Kamran Naraghi, Stephen P Tuck, Harish K Datta, Wan-Fai Ng, Jacob M van Laar

**Affiliations:** 1 Musculoskeletal Research Group, Institute of Cellular Medicine, Newcastle University, Newcastle-upon-Tyne, UK; 2 Department of Biochemistry, The James Cook University Hospital, Middlesbrough, UK; 3 Department of Rheumatology, The James Cook University Hospital, Middlesbrough, UK

## Background

The mechanism of osteoporosis in rheumatoid arthritis (RA) is multifactorial. However, inflammation and autoimmunity are key players in its pathogenesis. Treatment with tumour necrosis factor alpha (TNFα) blockers has been shown to be beneficial for bone metabolism and they were able to prevent bone deformities in RA. Moreover, they improved the bone mineral density (BMD) [1]. However, the role of B lymphocytes in bone turnover is still controversial. Mice models showed that B cell deficiency resulted in marked osteopenia [2]. This was explained by the marked decrease in bone marrow levels of osteoprotegerin (OPG) produced by B cells. However, treatment of 46 RA patients with rituximab revealed a marked reduction of the bone resorption marker beta-crosslaps (βCTX) after 6 months [3].

## Materials and methods

The HORUS trial aims to determine the effect of B cell depletion on bone turnover using bone markers and bone mineral density (BMD) measurements and to correlate these changes with circulating B cells in RA patients treated with rituximab. Forty-five RA patients are assessed every 3 months for one year following the administration of their first infusion of rituximab using clinical examination, BMD measured by DXA (primary endpoint), bone formation markers (BAP, OPG, P1NP, DKK1 and sclerostin), resorption markers (TRAP5b and CTX) in addition to flow cytometry (FACS) of peripheral blood CD19+ cells including subsets, and immunoglobulins.

## Results

Forty-five RA patients have been recruited in this on-going trial in ten UK sites. Two patients have successfully completed the study so far. Six patients have been withdrawn from the trial. Three patients have been found to be osteoporotic so they had to be started on bisphosphonates. Two patients did not have an appropriate response to rituximab. One patient developed lymphoma. A 1% increase in BMD between baseline and 12 months is considered a significant improvement based on a meta-analysis on anti-resorptive agents in osteoporosis which concluded that a 1% gain in bone density of the spine was associated with a statistically significant reduction of 8% in non-vertebral fractures and a study in a large population cohort showing decline of bone density over time in RA patients not treated with anti-resorptive agents [4].

## Conclusion

The HORUS trial examines the effects of rituximab on markers of bone turnover and bone mass in RA. This will help determine whether B cells play a role in RA associated bone loss.
